# Acute myocardial infarction in patient without cardiac risk factors during emergence from general anesthesia: a case report

**DOI:** 10.1186/s40981-020-00353-4

**Published:** 2020-06-16

**Authors:** Thu Nguyen Dang, Nam Tran Hoai, Son Nguyen Viet, Tri Le Huu, Khuong Truong Van, Cuong Nguyen Manh, Tiep Tran Dac

**Affiliations:** Department of Anesthesia, Military Hospital 103, Vietnam Military Medical University, 261 Phung Hung Street, Ha Dong District, Hanoi, Vietnam

**Keywords:** Perioperative myocardial infarction, Non-cardiac risk factors, General anesthesia

## Abstract

**Background:**

Perioperative myocardial infarction is a rare but highly fatal complication, which often occurs in patients with poor preoperative cardiac conditions undergoing high-risk surgery. We report a case of acute myocardial infarction in a patient without cardiac risk factors during emergence from general anesthesia for removal of spinal screws.

**Case presentation:**

A 37-year-old, 60 kg, and 160 cm man, who had no history of cardiovascular diseases, underwent removal of loosen spinal plug screws at L4-L5. The preoperative investigations revealed no abnormality and the patient was ASA I. The surgery was uneventful. During aspiration of the endotracheal tube, the patient suddenly experienced paroxysmal atrial fibrillation and ST segment elevation in DII lead. He was treated with oxygenation, optimal hemodynamics, minimize cardiac work, antiarrhythmias, and anticoagulation. The clinical conditions improved. Sinus rhythm was regained after 24 h and discharged without complications.

**Conclusions:**

Myocardial infarction can occur suddenly and unexpectedly in patients without risk factors after a low-risk surgery in any period of general anesthesia. Close monitoring and prompt treatment with this condition is important for improving outcomes.

## Background

Perioperative myocardial infarction (MI) is rare but associated with high mortality and morbidity (1.5–42%) [1]. It is an emergency and anesthetic challenge, which often occurs in patients with poor preoperative cardiac conditions undergoing high-risk surgery [2]. We report a case of acute MI in a patient without cardiac risk factors during emergence from general anesthesia for surgical removal of spinal screws.

## Case presentation

**Fig. 1:**
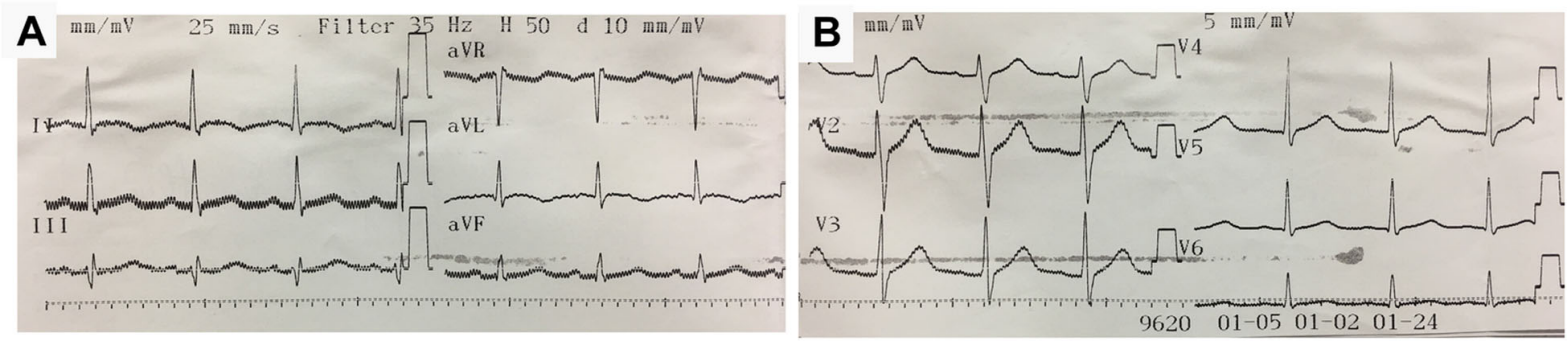
Preoperative 12-lead ECG of the patient: **a** DI-DIII, aVR, aVL, and aVF lead; **b** V1-V6 lead showed normal sinus rhythm and no sign of myocardial ischemia with HR 91 bpm, PR 156 ms, QRS 102 ms, and QT/QTc 398/446 ms

A 37-year-old, 60 kg, and 160 cm man was diagnosed with loosening of fixation screws to L4-L5 and scheduled for surgical removal under general anesthesia. He was otherwise well, without history of cardiovascular disease, diabetes, dyslipidemia, smoking, or chest pain. The last general anesthesia for screw implantation procedure was uneventful. Preoperative electrocardiography (ECG) showed normal sinus rhythm and no sign of myocardial ischemia (Fig. 1); chest X-ray was unremarkable; blood tests showed hemoglobin 14.7 g/dL and hematocrit 43.8%; and other laboratory findings were within normal ranges except for slightly elevation of aspartate aminotransferase (51 IU/L). The patient was American Society of Anaesthesiologists’ (ASA) classification I.

The patient was monitored during anesthesia, using ECG, saturation of peripheral oxygen (SpO_2_), and noninvasive arterial blood pressure. Preoperative blood pressure (BP) was 130/87 mmHg, heart rate (HR) 75 bpm, body temperature 36.1 °C, and SpO_2_ 98%. General anesthesia was induced and maintained with fentanyl (total 350 μg), propofol 120 mg, rocuronium 50 mg, and sevoflurane (1.5–2.5%). The surgery went uneventfully throughout the whole of 60 min of operation, with the patient on prone position, estimated blood loss of 100 mL, and without any complications. No significant changes in respiratory, cardiovascular, or neurological parameters was noted through the surgery.

Upon completion of the surgery, the patient was changed to the supine position. Ten minutes after discontinuation of sevoflurane, the patient was awake with HR 86 bpm, BP 135/85 mmHg, SpO_2_ 100% on spontaneous breathing, and ready for extubation. During endotracheal tube (ETT) aspiration, ECG suddenly changed with paroxysmal atrial fibrillation (AF) and fast ventricular response (HR 170–180 bpm), ST segment elevation (0.4–0.5 mV, max 0.6 mV), and BP decreased to 105/54 mmHg. Immediate setup of invasive hemodynamic monitoring lines showed arterial pressure of 85/40 mmHg and central venous pressure of 8 mmHg.

**Fig. 2:**
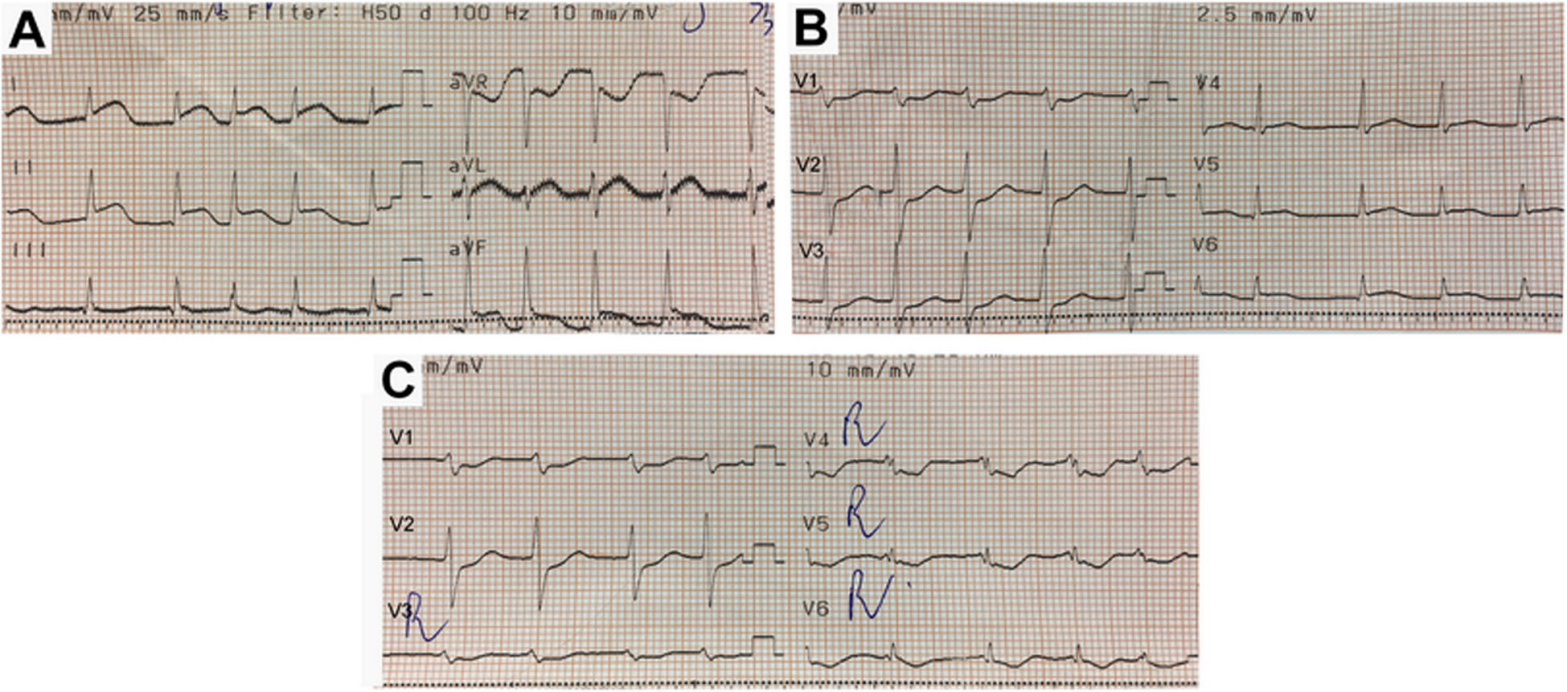
12-Lead ECG at 10 min after onset of ST elevation: **a** DI-DIII, aVR, aVL, and aVF lead; **b** V1-V6 lead; **c** V1, V2, and right precordial leads (V_3_R, V_4_R, V_5_R, and V_6_R) showed ST elevation in DI, DII, aVF, and ST depression in V1, V2, aVR, and right precordial leads

The patient was immediately sedated and mechanically ventilated with midazolam and fentanyl. Intravenous heparin (bolus of 4000 IU, then infusion at 600 IU/h) and amiodarone (bolus 150 mg, then infusion at 5 mg/kg/h) were given. Noradrenaline 0.03–0.1 μg/kg/min was infused to maintain the mean arterial pressure (MAP) of 60–70 mmHg. Transthoracic echocardiography (TTE) showed normal left ventricular wall motion and dimension (LVEDd 41 mm), with left ventricular ejection fraction of 57%. There was no right ventricular strain, pericardial effusion, or other pathology. 12-Lead ECG at 10 min after onset of ST elevation showed AF, ST elevation in DI, DII, aVF, and ST depression in V1, V2, aVR, and right precordial (V_3_R, V_4_R, V_5_R, and V_6_R) leads (Fig. 2). Arterial blood gas analysis showed mild metabolic acidosis (pH 7.38; PaCO_2_ 30 mmHg; HCO_3_− 18 mmol/L; BE − 6 mmol/L), normal serum electrolytes.

Tachycardia persisted with elevated ST segment during 10 min. Subsequently, HR- and ST-segment elevation gradually decreased (Table 1), and the noradrenaline dose was also slowly decreased.

**Table 1:** Hemodynamic parameters and ECG change after the onset of ST elevation

	10 min	30 min	1 h	4 h	16 h	24 h	48 h
Heart rhythm	AF	AF	AF	AF	AF	Sinus rhythm	Sinus rhythm
Heart rate (bpm)	160–170	120–130	90–100	90–100	90–100	80–90	70–80
Blood pressure	105/50	110/55	115/54	135/80	130/75	135/80	125/70
ST elevation DII (mV)	0.4–0.5	0.2–0.3	0.1–0.2	< 0.1	< 0.1	< 0.1	< 0.1

Trends in heart rhythm, heart rate blood pressure, and ST elevation of the patient after onset of ST elevation. *AF* atrial fibrillation

**Fig. 3:**
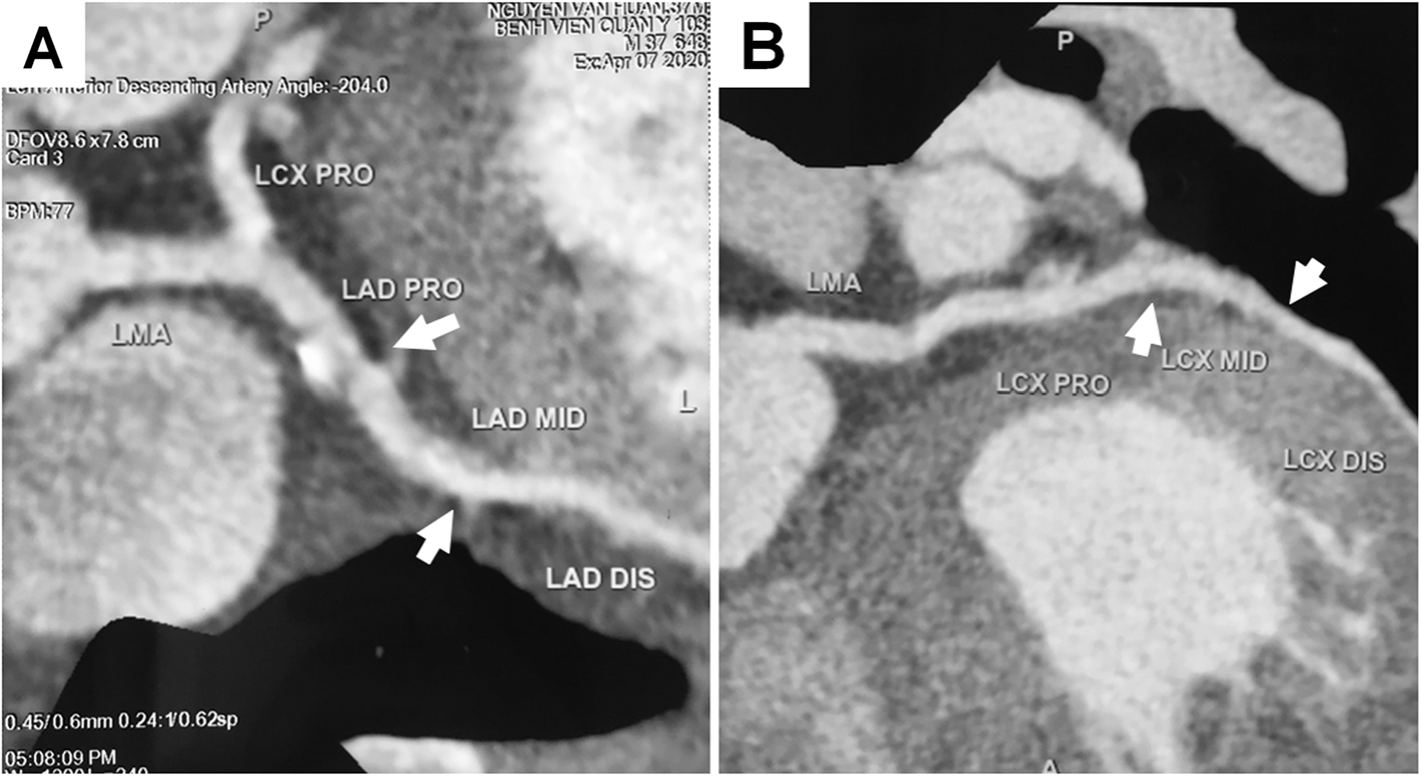
Postoperative CT coronary angiogram. Mild stenosis was noted at the middle left anterior descending coronary artery (LAD) (50%) and middle left circumflex coronary artery (LCx) (50%). LMA left main artery, PRO proximal, MID mild, DIS distal

After 4 h in the operating room, the patient was hemodynamically stable without noradrenaline. He was back on spontaneous breathing and transferred to the intensive care unit (ICU). Extubation was performed several hours later. Subsequent laboratory investigations showed elevation of Troponin-I and creatine kinase MB (CK-MB) levels (Table 2). Computed tomography coronary angiography showed the stenosis in the middle left anterior descending (LAD) (50%) and the middle circumflex coronary artery (LCx) (50%) (Fig. 3). Repeated TTE in ICU showed no significant wall motion abnormality. The heart returned to sinus rhythm, and HR and MAP were within normal limits after 24 h. The patient remained stable with normal sinus rhythm, chest pain-free, and downtrend in troponin-I and CK-MB levels. The patient was bridged to dual antiplatelet therapy with clopidogrel 75 mg and aspirin 100 mg daily. He was transferred to a general ward after 7 days in ICU and discharged on dual antiplatelet therapy at postoperative day 10 without any complications.

**Table 2:** Myocardial biomarkers change after the onset of ST elevation

	30 min	4 h	7 h	18 h	34 h	54 h
Troponin - I (pg/ml)	< 10	1723.8	2631.1	3602.9	1399.2	627.7
CK-MB (IU/l)	21	32.9	38.6	35.8	25.8	118.2

Normal range: Troponin - I (0.0–35 pg/ml); CK-MB (0–24 IU/l). *CK-MB* creatine kinase MB

## Discussion

Myocardial infarction is defined as an irreversible necrotic state of myocardium [3]. Most commonly ST elevation MI caused by an acute occlusion of a coronary blood vessel secondary to acute plaque rupture, thrombosis, or coronary artery vasospasm [3]. Perioperative MI often occurs in patients with poor preoperative cardiac conditions, such as advanced ages, smoking, hypertension, obesity, high cholesterol, diabetes mellitus, or family history of coronary artery disease [2, 4]. However, it may suddenly happen in a young patient without noticeable premedical history of coronary artery disease during emergence from general anesthesia for a relatively short and uneventful surgery. No known risk factor of coronary artery disease was reported in our patient. His pre-and intraoperative ECG did not show any sign of myocardial ischemia. Of note, invasive coronary artery imaging techniques were not indicated for this patient due to his young age and chest pain-free.

According to the Third Universal Definition of MI, acute MI is diagnosed based on the rise of cardiac biomarkers and at least one of the following criteria: symptoms of ischemia, ECG changes, changes on an echocardiography, or evidenced by angiography/autopsy [3]. Our patient was diagnosed of MI due to a significant elevation of Troponin I and CK-MB levels (Table 2) and new significant ST changes. In the settings of general anesthesia, ECG changes could be the only early sign of ischemia [3]. As such, close monitoring of ECG and hemodynamics is important to recognize MI during emergence from general anesthesia. While waiting for further investigations to confirm MI, prompt treatment should be initiated if MI is suspected [5]. Oxygenation, hemodynamic optimization, anti-arrhythmias, anticoagulation, coronary vasodilation, and mechanical ventilation to minimize myocardial oxygen consumption should be considered wherever appropriate [6, 7]. Coronary revascularization may be necessary if there is significant coronary stenosis. Since moderate stenosis of LAD (50%) and LCx (50%) were only lesions found in this patient, his acute MI could be associated with coronary vasospasm [8].

Coronary artery spasm could result from the interaction of vasoconstrictor stimuli and underlying abnormality of a coronary artery (endothelial dysfunction or a primary hyperreactivity of vascular smooth muscle cells), the causes of which is still unlear [9]. Potential general mechanisms to explain the occurrence of coronary vasospasm include redistribution of blood flow, altered humoral factors, increased catecholamine, and imbalance of vasoconstrictor-vasodilator forces. ETT aspiration causes stimulation of the vagus nerve and release of acetylcholine, which in turn cause release of norepinephrine from the postganglionic sympathetic nerve terminals in the heart, leading to excess stimulation adrenergic system [10]. Since large coronary arteries are innervated predominantly with alpha (vasoconstrictor) adrenergic receptors. ETT stimulation may trigger significant coronary vasoconstrictor. The other factors during emergence from general anesthesia, including pain, hypoxia, and inflammation, may increase the sensitivity of the coronary arteries to those stimuli.

Perioperative MI may be complicated with various types of arrhythmia, including AF (6–21%) [11] or even life-threatening ventricular arrhythmias. Arrhythmias, if not timely identified and treated, may worsen hemodynamic consequences caused by MI. Therefore, close monitoring of ECG and appropriate response to arrhythmias are required all period of general anesthesia to reduce mortality and morbility.

## Conclusions

Myocardial infarction can occur suddenly and unexpectedly in patient without risk factors after a low-risk surgery in any period of general anesthesia. Close monitoring of hemodynamics and ECG is very important for early diagnosis of this condition. If acute MI is suspected, prompt diagnosis and management may limit damages to myocardium and improve outcomes.

## Data Availability

Data relevant to this case report are not available for public access because of patient privacy concerns but are available from the corresponding author on reasonable request.

## Abbreviations

ASA: American Society of Anesthesiologists Classification
MI: Myocardial infarction
ECG: Electrocardiography
SpO2: Saturation of peripheral oxygen
BP: Blood pressure
HR: Heart rate
ETT: Endotracheal tube
AF: Atrial fibrillation
CVP: Central venous pressure
MAP: Mean arterial pressure
TTE: Transthoracic echocardiography
LVEDd: Left ventricular end-diastolic diameter
CK-MB: Creatine kinase MB
LAD: Left anterior descending artery
LCx: Circumflex coronary artery
ICU: Intensive care unit
